# Developing and Evaluating an Innovative Structural Competency Curriculum for Pre-Health Students

**DOI:** 10.1007/s10912-017-9449-1

**Published:** 2017-06-01

**Authors:** JuLeigh Petty, Jonathan M. Metzl, Mia R. Keeys

**Affiliations:** 1 Center for Medicine, Health, and Society, Vanderbilt University, Nashville, TN USA; 2 Department of Sociology, Vanderbilt University, Nashville, TN USA

**Keywords:** Structural competency, Premedical education, Health disparities, Race, Health justice

## Abstract

The inclusion of structural competency training in pre-health undergraduate programs may offer significant benefits to future healthcare professionals. This paper presents the results of a comparative study of an interdisciplinary pre-health curriculum based in structural competency with a traditional premedical curriculum. The authors describe a new evaluation tool, the Structural Foundations of Health Survey © (2016), developed to evaluate structural skills and sensibilities. The authors use the survey to evaluate two groups of graduating seniors at Vanderbilt University—majors in an interdisciplinary pre-health curriculum titled Medicine, Health, and Society (MHS), and premed science majors—with particular attention to understanding how political, cultural, economic, and social factors shape health. Results suggest that MHS majors identified and analyzed relationships between structural factors and health outcomes at higher rates and in deeper ways than did premed science majors. MHS students also demonstrated higher understanding of structural and cultural competency in their approaches to race, intersectionality, and racial health disparities. The skills that MHS students exhibited represent proficiencies increasingly emphasized by the MCAT, the AAMC, and other educational bodies that, in an era of epigenetics and social determinants, emphasize how contextual factors shape expressions of health and illness.

Structural competency has emerged as an important new framework for understanding relationships between medical and social domains. Structural competency (Metzl 2010; Metzl and Hansen 2014; Metzl and Roberts 2014) builds on cultural competency to provide a framework that imparts to medicine the notion that matters of race, ability, sexual orientation, socioeconomic class, or other markers of difference shape interactions between patients and doctors. Whereas cultural competency focuses mainly on identifying clinician bias and improving clinical communication, structural competency emphasizes diagnostic recognition of the economic and political conditions that produce health inequalities in the first place. Structural competency calls on healthcare providers not only to recognize how institutions, markets, or healthcare delivery systems shape symptom presentations but also to mobilize for correction of health and wealth inequalities in society.

Given this framing, most structural competency interventions have targeted healthcare providers and medical students. As Metzl and Hansen (2014) describe it, “if stigmas are not primarily produced in individual encounters but are enacted there due to structural causes, it then follows that clinical training must shift its gaze from an exclusive focus on the individual encounter to include the organization of institutions and policies, as well as of neighborhoods and cities, if clinicians are to impact stigma-related health inequalities” (127). Thus, medical educators at the State University of New York at Albany developed a structural competency webinar for healthcare providers (School of Public Health), structural competency programs have been developed for nurses (Garcia 2015), and structural competency electives emerged in a number of U.S. medical schools (Pérez 2014).

This paper assesses ways that structural competency training is beneficial in pre-health baccalaureate settings as well and in ways that might enhance traditional pre-med education. Pre-health students learn a great deal about the biological aspects of illness but traditionally receive less training regarding the social and economic structures that produce inequities in the distribution of these illnesses. Instruction in these latter issues becomes increasingly important as developments in economics, sociology, the medical humanities, urban planning, epigenetics, and neuroscience uncover the vital roles that social contexts play in even the most seemingly biological of illnesses (Johnstone and Baylin 2010; Slopen et al. 2014), and as educational bodies such as the AAMC (Englander et al. 2013; Association of American Medical Colleges 2014) and the MCAT (Association of American Medical Colleges 2015; Schwartzstein 2013) increasingly emphasize recognition of the “social foundations” of health.

Honing this knowledge during the undergraduate years becomes an ever more significant and applicable skill set for the next generation of health practitioners and providers. For instance, in the new MCAT, medical students are expected to demonstrate competency in the influences of culture and community on health behaviors and outcomes, basics of the U.S. healthcare system, social determinants of health, and changes in health policy (Association of American Medical Colleges 2011). Medical school admission requirements frequently echo these curricular changes. In addition to the natural sciences, recent AAMC core competencies for premedical students include understanding the psychological and socio-cultural influences on health, cultural competency, and skills such as critical thinking, teamwork, oral communication, and written communication (Association of American Medical Colleges 2014).

But, how is it possible to translate structural competency frameworks developed for health professionals for use into semester-long baccalaureate courses? And what are the most effective ways to evaluate pre-health students’ grasp of concepts central to structural competency such as structural inequity, structural racism, or structural stigma?

These questions undergirded the expansion of an innovative interdisciplinary pre-health baccalaureate curriculum at Vanderbilt University in Nashville, Tennessee. The curriculum, titled Medicine, Health, and Society (MHS), arose in response to student demand for courses that explored the social and cultural aspects of health and illness. In its first iterations, the MHS major combined health sciences classes with a small number of university courses in the medical humanities and medical social sciences.

Demand was remarkable. Student enrollment rose from 172 student majors in 2008 to over 300 majors in 2011. In response to this growth, MHS faculty met over the course of 2012-13 to revise the curriculum in ways that brought medical humanities approaches such as narrative analysis and critical thinking into conversation with social scientific attention to cross-cultural, racialized, and gendered determinants of health (Vanderbilt University Center for Medicine Health and Society 2015). Structural competency emerged as a central rubric in this curricular reformulation because of its inherent interdisciplinarity.

The revised 36-credit-hour MHS major (Table 1) allowed students to choose from one of six concentration areas. The curriculum also added a series of new medical humanities courses on racial and ethnic health disparities; structural aspects of mental health, politics of health, health activism, disability studies, critical perspectives on global health, and interdisciplinary research methods. So too, existing medical humanities courses added structural immersion assignments that explored literary tensions regarding health and illness alongside exercises that asked students to, for instance, critically engage with ways that such structural frameworks as food delivery systems, diagnostic categories, structural racisms, or urban architectures might impact plot developments (Table 1).

**Table 1:** Medicine, health and society (MHS) curriculum overview

MHS Major Requirements^a^		Examples of MHS course titles and content
One core course	• Politics of health • Racial & ethnic health disparities • Fundamental issues in MHS • Theories of the body • American medicine in the world • Masculinity and men’s health	*Racial and Ethnic Health Disparities* addresses historical, cultural, institutional, economic and political factors that shape health disparities in the U.S. Students evaluate strategies to eliminate the disparity and address the root structural cause of the health outcome. *Narrative Medicine* explores the role of narrative to increase understanding of patients, the patient experience, and medical knowledge. Students develop their writing and learn about a variety of illness narratives and theoretical approaches in narrative medicine. *Politics of Health* addresses U.S. health policy and political dimensions of various health-related issues. Students critically evaluate the content of research, popular press articles, and websites. *Medicine and Literature* explores health, illness, and identity through works of literature. Students learn textual analysis and writing skills. *Global Health and Social Justice* examines global health institutions, policies, and practices. Students learn critical and cultural theory anthropological, sociological, and scientific approaches to global health problems. *Designing Healthy Publics* studies how buildings, cities, and urban planning structure the health of populations, and a number of classes on race, ethnicity, and health explored ways that historical, cultural, institutional, economic and political factors shaped patterns of morbidity, food distribution networks, medication reimbursement rates, injury patterns, and other factors.
Four courses in one concentration area	• Global health • Health behaviors and health sciences • Health policies and economies • Inequality, intersectionality, and health justice • Medicine, humanities, and the arts • Critical Health Studies^b^	
One Disciplinary Course^c^	• Health economics • History of medicine • Sociology of medicine • Medical anthropology • Women’s health • Health psychology	
Six elective courses	As part of their electives, students may take up to 4 biomedical prerequisites.	

^a^Each course counts as 3 credit-hours toward the 36-credit-hour major in MHS.

^b^The Critical Health Studies allows students to craft an individualized plan of study with a MHS faculty adviser.

^c^The Disciplinary Course is a distribution requirement; it may be taken as part of the concentration or elective courses.

By 2013, Medicine, Health, and Society became the fastest growing and third-largest undergraduate major on a campus of roughly seven thousand undergraduates, and in 2015 the major enrolled more than 500 undergraduate majors. Pertinent to this paper, a majority of Vanderbilt students meanwhile continued to pursue traditional pre-health degrees as pathways to professional schools. Most premed students majored in interdisciplinary sciences such as neuroscience, molecular and cell biology, biomedical engineering, or other courses of study that emphasize life sciences along with smaller numbers of required general education courses in the humanities and social sciences (Baum and Rains 2014).^.^

The divergence of two pre-health tracks at the same school—one (premed) that emphasized the traditional sciences, another (MHS) that promoted cultural and cross-cultural analysis alongside science medical school prerequisites—allowed us to measure whether a curriculum based in structural competency might impart different skills than did traditional premed tracks, while at the same time preparing students for their post-college careers. Preliminary data validated the consistency of the evaluation plan and suggested that MHS majors competently identified and analyzed structural foundations of health (Metzl and Petty 2017) This paper compares the outcome of the MHS curriculum emphasizing structural competency with a traditional premedical curriculum emphasizing the sciences.

## Methods

To compare MHS and traditional pre-health students’ cultural and structural competency skills, we devised an evaluation instrument to assess recognition of structural issues including understanding ways that political, cultural, economic, and social factors shape health outcomes. The Structural Foundations of Health Survey © (2016) begins with questions about professional preparation, structured as Likert scale questions about healthcare-specific knowledge and general academic skills based on the AAMC’s “Core Competencies for Entering Medical Students” (Association of American Medical Colleges 2014). We next adapt the Attributional Complexity Scale (Fletcher et al. 1986), which consists of fourteen items designed to measure explanations of behavior based on consideration of external and internal factors. Sample items include, “I think a lot about the influence that society has on my behavior and personality,” and “I don't usually bother to analyze and explain people's behavior.” Previous research has identified associations between attributional complexity, critical thinking skills and ability to detect bias (Reid and Foels 2010). The survey then includes five open-ended questions which ask respondents to: 1) list the three most important influences on people’s health; 2) identify the three most important tensions in a cross-cultural physician-patient vignette; 3) choose and explain the three most important factors that explain disparities in childhood obesity; 4) choose and explain the three most important factors that explain disparities in heart disease mortality rates in men; and 5) describe messages about depression reflected in a pharmaceutical advertisement for an antidepressant medication.

We modeled the open-ended question about factors that influence health based on focus group research conducted by the Robert Wood Johnson Foundation (RWJF) and reported in *A New Way to Talk about the Social Determinants of Health* (Carger and Westen 2010). In the RWJF research, only a small fraction of respondents addressed social determinants when asked about the influences on health, but more respondents recognized social determinants of health as important when prompted with examples. Therefore, in our survey, this question preceded case-based questions about health disparities to test for recognition of social determinants without prompting. Our first case was a vignette adapted from case studies commonly used in cultural competency training and assessment:

*Mrs. Demetilla Hernandez is a 63-year-old Cuban woman who went to the HMO clinic because of weakness and fatigue for the last two months…Because Mrs. Hernandez cannot speak English, the family language at home is Spanish… …Mrs. Hernandez became a little agitated, explaining to her daughter that she thought that the traditional Cuban dishes she prepares are healthy…*

Three other cases covered childhood obesity, heart disease, and pharmaceutical advertising. For childhood obesity, we showed a map of the U.S. from a 2009 *Trust for America's Health* report (2010), which showed that the U.S. South contained eight of the ten states with the highest rates of childhood obesity. For heart disease, we cited a statistic that, “African-American men are 30% more likely to die from heart disease than non-Hispanic white men” (U.S. Department of Health and Human Services 2015). The pharmaceutical advertisement depicted a woman who appeared to be white and middle-aged, who smiled while holding up a white-diapered infant above text that read, “I got my playfulness back!” Through this collection of cases, the Social Foundations of Health survey assesses ability to analyze physician-patient encounters, health disparities, and media using the tools of structural competency.

We conducted one-way analysis of variance to analyze the effects of the pre-health curriculum on students’ ability to recognize social determinants of health, and paired t-tests were used to compare group means on Likert-scaled items. For the open-ended responses, the authors independently read all of the responses and then met to create codes for each set of responses. Responses were independently coded by a trained PhD student and confirmed among the authors. Agreement was .92.

MHS seniors completed the survey online as part of their graduation exam. Premedical students not majoring or minoring in MHS were recruited through a study announcement with a link to the anonymous online survey emailed to all students on Vanderbilt’s Health Professions Advisory Office (HPAO) listserv. Premed participants received $20 Amazon gift certificates. We administered the study in spring 2015. Average completion time was 30 min. The Vanderbilt University IRB granted exempt status for this study.^1^

## Results

Of the 177 students who completed the survey in spring 2015, 155 students (85 MHS majors out of a potential pool of 127; and 70 non-MHS premed majors out of a pool of 168) remained in the sample after we excluded incomplete surveys. 42% (*n*=36) of the MHS respondents identified as premed. Most of the remaining MHS students planned careers in nursing, public health, healthcare administration, humanities, and consulting.^2^

While most students reported “good” professional preparation (Table 2), MHS majors reported better preparation than premed majors regarding understanding the relationships between socioeconomic status and health, ability to discuss controversial issues, knowledge of the U.S. healthcare system, and knowledge of the Patient Protection and Affordable Care Act (ACA).

**Table 2:** Self-reported professional preparation^a^

	Respondents, mean (sd)			
Indicate how well you were prepared in each area through your program of study^b^	Total (*n*=148)	MHS (*n*=85)	Premed (*n*=63)	*p*-value
Oral communication skills	3.5 (1.03)	3.54 (.97)	3.49 (1.11)	.775
Understanding of the relationship between socioeconomic factors, health, and medicine	4.1 (1.17)	4.67 (.61)	3.33 (1.31)	<.001
Overall knowledge about the American health system	3.043 (1.19)	3.55 (.98)	2.37 (1.11)	<.001
Ability to work cooperatively with diverse people	4.18 (.99)	4.29 (.86)	4.03 (1.14)	.111
Knowledge of basic components of the Affordable Care Act	2.76 (1.30)	3.19 (1.19)	2.17 (1.23)	<.001
Writing ability	4.00 (.94)	4.11 (.87)	3.87 (1.01)	.135
Research skills including formulating research questions and hypothesis	4.02 (.94)	3.93 (.92)	4.16 (.95)	.143
Critical thinking skills	4.41 (.74)	4.35 (.72)	4.50 (.76)	.207
Ability to discuss and negotiate controversial issues	4.25 (.86)	4.41 (.76)	4.03 (.95)	.008
Interpreting quantitative research	4.04 (1.02)	4.01 (.99)	4.08 (1.07)	.692

^a^Data are from 155 seniors who responded to a spring 2015 survey about the pre-health professional curriculum

^b^Students indicated their professional preparedness in each of these areas on a scale of 1 (poor preparation) to 5 (excellent preparation)

Both MHS and premed students scored equally well on the attributional complexity scale, suggesting similar aptitudes toward complex explanations of human behavior (Fletcher et al. 1986) and ability to detect bias (Reid and Foels 2010).

**Fig. 1:**
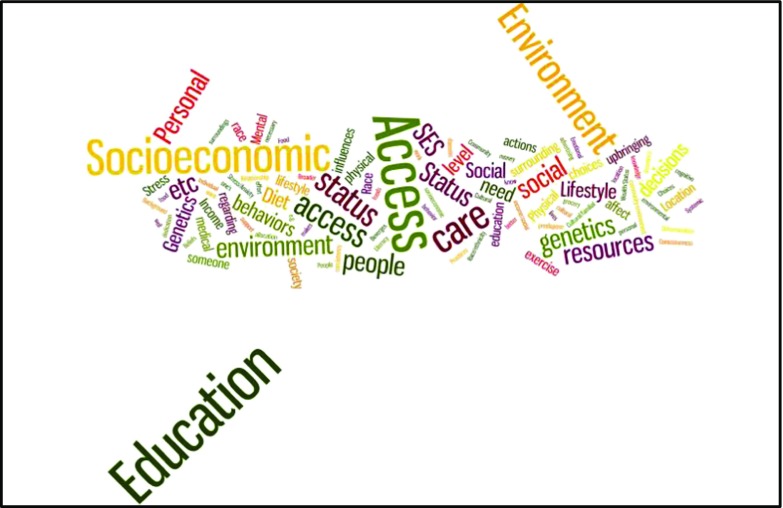
Word map of MHS majors’ open-ended response to “What are the three most important influences on people’s health?’’. Data are from 155 seniors who responded to a spring 2015 survey about the pre-health professional curriculum

**Fig. 2:**
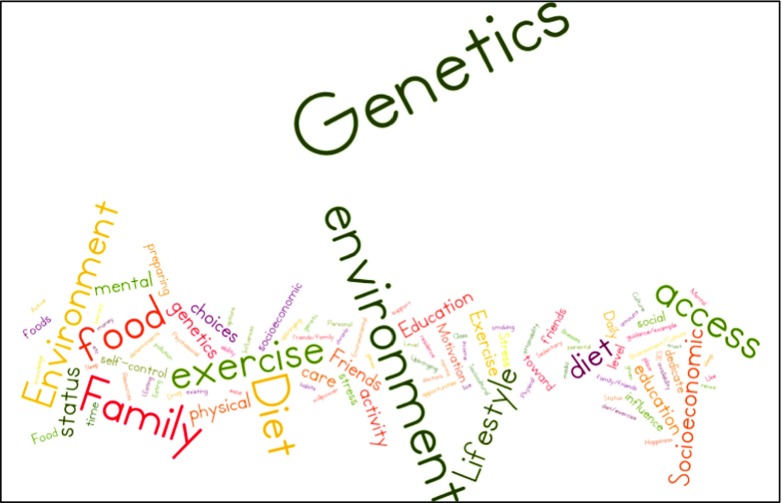
Word map of premed majors’ open-ended response to “What are the three most important influences on people’s health?’’. Data are from 155 seniors who responded to a spring 2015 survey about the pre-health professional curriculum

We observed important differences in students’ responses to the question, “What are the three most important influences on people’s health?” MHS students were significantly more likely to list structural factors, as demonstrated by word clouds of each group’s response (Figs. 1 and 2).^3^ According to ANOVA analysis of the top ten responses, MHS majors were more likely to identify socioeconomic status (MHS: 36/85[42%]; premed: 12/70 [17%], *p*<.001) and environmental or societal factors (MHS: 41/85[59%]; premed: 27/70 [39%], *p*<.05). Premed students were more likely to identify individual factors (MHS: 13/85[19%]; premed: 33/70 [47%], *p*<.001).

As part of their analysis of the “Mrs. Hernandez” vignette, students were asked what additional information was needed to address her case and to suggest three important questions. We identified and coded the ten most common questions. Both MHS and premed majors posed questions about Mrs. Hernandez’s finances and her ability to afford medication. However, as Table 3 details, premed majors consistently identified individual, clinical, or cultural level factors as the root of the problem, such as “cultural mistrust of physicians,” “cultural dietary practices,” or “language barriers.” Meanwhile, MHS students frequently identified structural explanations such as transportation and neighborhood.

**Table 3:** Illustrative quotes from open-ended responses (coded words underlined)^a^

Prompt:	MHS	Premed
Cross-cultural Vignette	Mrs. Hernandez may live in a food desert and be unable to obtain the necessary fresh vegetables and fruit to improve her diet…This isolation could negatively impact her adherence to her treatment plan Mrs. Hernandez may have refused to buy prescription medicines because she knows it would be a burden for her daughter because they don't have health insurance to cover the cost	Since Mrs. Hernandez is a foreigner, she may not necessarily trust or listen to an American doctor. Also, the language barrier may have caused Mrs. Hernandez to not listen to the doctor
Childhood Obesity US South	...income is strongly correlated with obesity, with lower income populations exhibiting higher rates of overweight and obesity than wealthier populations. Since many of the poorest states in the US are located in the South, income is an important factor... Finally, the South has a long history of racism, which is still present in the form of institutional racism...	Southern comfort food is a hallmark of the South. Greasy, fried, and buttered goods are a must. Therefore, I chose cultural background as one of the most important factors in contributing to the high obesity rates…Individuals dictate their own choices and lifestyle.
Cardiac Mortality	Neighborhood factors, such as a lack of sidewalks or community centers, may contribute to poor heart health among African American men there still exists a heavy degree of institutional racism that is found in conjunction with physician bias and lower socioeconomic status that affects access to healthcare.	I think there are many lifestyle differences between these two groups that contribute to most of the disparity If the problem is due to race, it is most likely genetic
Drug Ad	The ad caters to the beliefs that men do not have mental disorders, that women are the ones that should be caring for children, and that women need to be able to care happily and energetically for their children	This advertisement plays on the guilt of mothers who feel that their mental illness is making them neglect their children. It is therefore shaped by the social and cultural norms of America, where mothers are expected to be very cheerful around their children

^a^Data are from 155 seniors who responded to a spring 2015 survey about the pre-health professional curriculum

In response to questions about health disparities, participants selected three from a list of fourteen items that included AAMC cultural competency factors (cultural background, health literacy, health traditions and beliefs, physician bias) (Association of American Medical Colleges 2005), individual-level factors (genetics, individual lifestyle choices), and structural competency factors as defined by Metzl and Hansen (2014) (access to healthcare, health delivery system, insurance, institutional racism, medicalization, income, neighborhood, social policies). MHS majors were more likely to select one or more cultural factor (MHS: 75/85[89%]; premed: 55/70 [54%], *p*<.05) and less likely to select one or more individual-level factors (MHS: 25/85[29%]; premed: 34/70 [49%], *p*<.05) as one of the three most important factors explaining why childhood obesity rates are highest in the U.S. South (Table 4). MHS majors were also more likely than premed majors to select one or more structural factors (MHS: 78/85 [92%]; Premed: 56/70 [80%]; *p*<.05) among the three most important factors explaining disparities in heart disease.

**Table 4:** Students’ identification of three factors which best explain select health disparities^a^

	Respondents, no (%)							
	Childhood obesity				Heart disease			
Respondents selecting at least 1 from each category	Total (*n*=155)	MHS (*n*=85)	Premed (*n*=70)	*p*-value	Total (*n*=155)	MHS (*n*=85)	Premed (*n*=70)	*p*-value
*Cultural competency factors:* cultural background, health literacy, physician bias, health traditions and beliefs	130 (84)	75 (89)	55 (54)	.024	104 (67)	56 (52)	48 (69)	.947
*Structural factors:* access to healthcare, health delivery system, health insurance, institutional racism, income, neighborhood, social policies	117 (75)	72 (85)	45 (64)	.112	134 (86)	78 (92)	56 (80)	.012
*Biomedical, individual factors:* genetics, individual lifestyle	59 (38)	25 (29)	34 (49)	.017	55 (35)	31 (37)	24 (34)	1.00

^a^Data are from 155 seniors who responded to a spring 2015 survey about the pre-health professional curriculum

In the qualitative analysis of open-ended responses to the childhood obesity prompt (“explain the three most important factors that explain geographic disparities in childhood obesity”) we coded student responses for connections between health outcomes and cultural and structural factors. While most students addressed at least one cultural and structural factor, MHS students were more likely to explain geographic disparities in obesity as consequences of individual or family level factors (MHS: 56/85 [66%]; Premed: 28/70 [40%]; *p*=.001) and broader socio-political factors (MHS:47/85 [56%]; Premed: 20/70 [29%]; *p*<.001) (Table 5). MHS students were also more likely to link cultural differences to structural factors (MHS: 33/85 [39%]; Premed: 14/70 [20%]; *p*=.011) (Table 5). For instance, premed students frequently linked “cultural” explanations to individual life choices (Table 3). Meanwhile, though MHS students commonly identified cultural factors, they were far more likely than premed students to causally link structural factors to health outcomes.

**Table 5:** Students’ open-ended responses explaining health disparities^a^

	Respondents, no (%)							
	Childhood obesity				Heart disease			
Demonstrated competencies:	Total (*n*=155)	MHS (*n*=85)	Premed (*n*=70)	*p*-value	Total (*n*=155)	MHS (*n*=85)	Premed (*n*=70)	*p*-value
*Cultural competency, Basic.* Identify key cultural issues identified by the AAMC that may impact patient behavior and decision-making: language/interpreter, religion, spirituality, trust, health beliefs and attitudes, health traditions, health literacy, food traditions, family	118 (76)	71 (84)	47 (67)	.017	53 (34)	23 (27)	30 (43)	.039
Link cultural difference to structural contexts identified by the AAMC: social determinants of health (e.g. SES, neighbor factors, cost of healthcare), health systems, and institutional racism.	47 (30)	33 (39)	14 (20)	.011	11 (7)	8 (9)	3 (4)	.219
Link health outcomes to individual or family level factors that impact living and working conditions: income, educational level, health insurance status, and access to healthcare.	84 (54)	56 (66)	28 (40)	.001	64 (42)	38 (45)	26 (37)	.344
*Social/political.* Link health outcomes to broad social, political and economic factors: neighborhood environment, racism, health delivery system, and health policy.	68 (44)	48 (56)	20 (29)	<.001	30 (19)	16 (19)	14 (20)	.855
*Race/ethnicity.* Demonstrate understanding of the relationship between race and health as an outcome of cultural and social factors including:								
Physician bias	9 (6)	4 (5)	5 (7)	.522	46 (30)	40 (47)	6 (9)	<.001
Discrimination	11 (7)	8 (9)	3 (4)	.219	31 (20)	26 (31)	5 (7)	<.001
Institutional Racism	10 (6)	6 (7)	4 (6)	.737	64 (41)	43 (51)	21 (30)	.009
Socioeconomic Differences	13 (8)	7 (8)	6 (9)	.941	22 (14)	21 (25)	1 (1)	<.001

^a^Data are from 155 seniors who responded to a spring 2015 survey about the pre-health professional curriculum.

Students’ explanations of disparities in heart disease also demonstrate variations in depth of analysis (Tables 3, 5), particularly regarding race. While MHS and premed majors were equally likely to address race in their explanations (MHS: 51/85 [60%]; Premed: 37/70 [53%]; *p*=.375), MHS students were more likely to define racial disparities as the consequence of socioeconomic differences, discrimination or stereotypes, or of policies that had racial consequences (Table 5).

Both groups were equally likely to provide medical responses to the drug advertisement (e.g. “Depression is a serious illness and/or depression can be treated with prescription pharmaceuticals”). MHS students were slightly more likely than premed majors to critique the pharmaceuticalization of mental illness (MHS: 32/85 [38%]; Premed: 15/70 [21%]; *p*= .029). Qualitative analysis of responses to the open-ended questions asking students to identify messages about depression and other social phenomena in the ad revealed equally low levels of critical engagement with race, class, and gender: only 5% of all students addressed the woman’s race (white) and 1% addressed her class. The gender analysis was somewhat better with 23% of all students addressing the gendered portrayal of depression in their responses.

## Discussion

Our pilot data suggest that students who graduated from an interdisciplinary pre-health curriculum (MHS) identified and analyzed relationships between structural factors and health outcomes at higher rates and in deeper ways than did premed science majors while simultaneously attaining comparable rates of admission to medical schools.^4^ While both groups demonstrated high levels of awareness of the impact of cross-cultural factors on health outcomes, MHS majors demonstrated advanced skills that implied more nuanced understandings of structures underlying illness and health. For instance, MHS students more frequently listed structural or institutional racism as an explanatory factor for racial, economic, and demographic disparities and more commonly defined these disparities as arising from socioeconomic differences, discrimination, or policies that resulted in intended or unintended racial consequences.

Our study has certain limitations. For logistical^5^ reasons we were only able to evaluate students at the distal points of their baccalaureate degrees. Therefore, a subsequent study will compare skills of first-year students and graduating seniors. Our initial survey focused on health disparities in the U.S. and did not at this point assess recognition of the structural aspects of global health. And, since we focused on the skills and frameworks imparted by interdisciplinary medical humanities, we did not assess the complex worldviews and methodologies that premed science majors gain in advanced science courses.

At the same time, our data suggest that structural competency is a skill set that develops not just from attitudes, but also from training. Indeed, both groups of students demonstrated similar levels of sensitivity to cultural biases. Yet, students trained in interdisciplinary and intersectional methods showed enhanced ability to diagnose and analyze issues such as determinants of health, structural stigma, health economics, and race, while at the same time also demonstrating deeper understandings of the “cultural” components of cultural competency. The one exception was recognizing whiteness as a racial category; this influence on health remained invisible and uncritically assumed by both groups.

It may well be argued that MHS students simply reproduced the structural language and analysis emphasized by their coursework—but this is in part the point. The skills that these students demonstrated represent ones increasingly accentuated by the MCAT, the AAMC, and other bodies that recognize, in an era of epigenetics and social determinants, how contextual factors shape expressions of health and illnesses (Robert Wood Johnson Foundation 2015; Goldstein and Holmes 2011).

Further evidence suggests that these skills also represent ones that challenge physicians in clinical practice. Fully 85% of primary care providers and pediatricians polled in a recent RWJF survey agreed with the statement that “unmet social needs are leading directly to worse health for all Americans” while at the same time voicing concern that they did not “feel confident in their capacity to meet their patients’ social needs,” and that their failure to do so “impedes their ability to provide care” (Goldstein and Holmes 2011). Meanwhile, increasing numbers of physicians cite structural factors, such as restrictive insurance policies or lack of time with patients, as reasons to leave clinical practice (Pathman et al. 2002).

In this sense, our study also contributes to an evolving literature such as many of the other essays in this issue (Berry and Lamb 2017) suggesting that teaching students about the “social” aspects of medicine needs to begin sooner in the educational process, during the baccalaureate years. Ultimately, teaching students to recognize the social and structural contexts of health and illness depends not just on challenging implicit biases, but also on promoting methodologies and skill sets that can help future physicians take the lead in championing health justice and social change.

## Endnotes

- ^1^IRB# 150422, exempt status granted 3/23/15.
- ^2^To confirm that premed students had not completed the MHS curriculum, we asked them how many MHS courses they took as part of their undergraduate coursework. 61% (*n*=43) did not take any MHS courses; 7.1% (*n*=5) took just one course, 2.8% (*n*=2) took three to five courses, and 10% (*n*=7) could not remember. All of these students were included in the analysis.
- ^3^Open-ended responses are visually represented with figures generated by Wordle, an online tool that creates word clouds from input text. The text was cleaned up by removing filler words (“and”, “the”). The size of words in the clouds indicates word frequency.
- ^4^To be clear, not every MHS student applied to medical school. However, MHS medical school applicants were accepted at rates comparable to traditional premed students. According to 2014 data from the Health Professions Advisory Office (HPAO) (Baum and Rains 2014), medical school acceptance rates for the three most popular premed majors were Neuroscience (61%), MHS (62%), and Molecular & Cellular Biology (65%), compared to a national average of 43%.
- ^5^Many students wait until their junior years to formally declare their majors.
